# Hypoluteoidism in a dog associated with recurrent mammary fibroadenoma stimulated by progestin therapy

**DOI:** 10.1186/s13028-017-0324-x

**Published:** 2017-09-06

**Authors:** Maria Teresa Zedda, Luisa Bogliolo, Elisabetta Antuofermo, Laura Falchi, Federica Ariu, Giovanni Pietro Burrai, Salvatore Pau

**Affiliations:** 1 Section of Obstetrics and Gynecology, Department of Veterinary Medicine, University of Sassari, Via Vienna n.2, 07100 Sassari, Italy; 2 Section of Pathology, Department of Veterinary Medicine, University of Sassari, via Vienna n.2, 07100 Sassari, Italy

**Keywords:** Hypoluteoidism, Mammary fibroadenoma, Pregnant bitch, Progestin treatment

## Abstract

**Background:**

Hypoluteoidism in the bitch is characterized by insufficient production and secretion of progesterone by the corpora lutea. It is a rare pathologic condition and during pregnancy, it leads to embryonic resorption or fetal abortion. Supplementary therapy with progestins is indicated during pregnancy to obtain delivery of vital puppies but unwarranted side effects of such treatment are poorly documented.

**Case presentation:**

A 4-year-old, nulliparous, female Istrian Shorthaired Hound dog had been mated repeatedly in six heats with different dogs of proven fertility but signs of pregnancy did not develop. Estrous cycles, mating and pregnancies were monitored as hypoluteoidism or genital disease was suspected. During the first monitored estrus, the bitch was mated and on day 18 [day 0, day of estimated peak of luteinizing hormone (LH)], ultrasound examination showed three amniotic vesicles that were however found to be resorbed between day 20 and 23. Progesterone concentrations, measured by ELISA, were >8 ng/mL until day 12 and 1–2.5 ng/mL on days 20, 23 and 26. Primary hypoluteoidism was therefore suspected. In the second monitored estrus, the bitch was mated and during pregnancy, progesterone concentrations were >8 ng/mL until day 17 and 1–2.5 ng/mL on day 19. On days 20 and 22, two out of three embryonic vesicles had been resorbed. The bitch was treated with progesterone in oil from day 19 to day 58. Increase in the size of 2nd left thoracic mammary gland (T2-L) was observed and on day 46, ultrasound evaluation and biopsy were performed revealing a low-cellularity fibroadenoma. Parturition started spontaneously at day 65 but due to dystocia caused by fetal macrosomia, a Caesarean section was performed. During the next (third) monitored estrus, the bitch was bred again and during pregnancy, early decrease in progesterone concentration confirmed the diagnosis of primary hypoluteoidism. The bitch was treated with synthetic progestin (altrenogest) from day 8 to day 57. Five amniotic vesicles were detected by ultrasonography. Recurrence of swelling of T2-L was observed. On day 60, the bitch whelped five pups, two males and three females. As reported later by the owner, the latter did not show any sign of heat over the past 3 years. In one of them, clitoral hypertrophy and a blind ending vagina were diagnosed.

**Conclusions:**

This is the first description of early hypoluteoidism in a pregnant bitch developing a mammary fibroadenoma under progestin treatment.

## Background

Primary hypoluteoidism in bitches is characterized by insufficient production and secretion of progesterone by the corpora lutea. Plasma progesterone concentrations above 2.0–2.5 ng/mL (6.4–7.9 nmol/L) are necessary for maintenance of pregnancy and plasma concentrations below 2 ng/mL for more than 48 h are likely to cause embryonic resorption or abortion [1, 2]. The causes and the pathogenesis of canine hypoluteoidism are poorly understood and the diagnosis is often difficult to establish as the decline in progesterone levels may occur as a consequence of many conditions such as maternal illness, fetal death and infectious diseases [3–5]. Enzyme-linked immunosorbent assay (ELISA) for measuring blood progesterone are available and are providing clinicians an immediate and fairly accurate tool for the diagnosis of this pathologic condition [6, 7].

Progesterone deficiency in pregnant bitches has been treated with a variety of progestins including medroxyprogesterone acetate (MPA) [1, 8], altrenogest [7, 9] and progesterone in oil [10, 11]. Unwarranted effects of progestin supplementation, including masculinization of female fetuses have rarely been reported [12]. Progestins are a well known triggering cause of mammary gland fibroadenoma in cats (fibroepithelial hyperplasia or feline mammary fibroadenomatous hyperplasia complex) [13, 14], while this association has not been reported in dogs. Here, a case of hypoluteoidism in a pregnant bitch associated with embryonic mortality and development of a mammary gland fibroadenoma following progestin supplementation is reported.

## Case presentation

A 4-year-old, nulliparous, Istrian Shorthaired Hound dog of 19 kg body weight, was referred to the Veterinary Teaching Hospital (VTH) of the University of Sassari, Italy, with a history of infertility. The first estrus occurred when the bitch was 11 months old with an interestrus interval of five months. The bitch was mated repeatedly in six heats with different dogs of proven fertility but signs of pregnancy such as increase in abdominal volume, swelling of mammary glands and behavioral changes were not observed. The bitch had not previously received medical or hormonal treatments and other bitches in the kennel had normal fertility.

The first clinical examination, including inspection of vulva and vagina, abdominal palpation, vaginoscopy, ultrasound of the uterus (SA 600V; linear probe LV 4-7MH, Ge Medical Systems Kretztechnik, Teufenbach, Austria) and palpation of mammary glands was carried out on day 105 after the last mating and did not reveal any abnormalities. Cytological evaluation of a vaginal smear indicated anestrus. A vaginal swab was collected for bacterial culture revealing a normal saprophytic flora consisting of streptococci. The bitch was serologically negative for *Brucella canis,* canine herpesvirus*, Ehrlichia canis, Rickettsia* spp*., Bartonella henselae, Toxoplasma gondii, Neospora caninum* and *Leishmania* spp. Differential diagnosis included hypoluteoidism and genital abnormalities such as segmental uterine or tubal aplasia or acquired atresia resulting from inflammatory processes. In order to determine the cause of infertility, the subsequent estrous cycles and pregnancies were carefully monitored. A vaginal smear characterized by a shift from cornified superficial cells to reappearance of intermediate and parabasal cells was considered as characteristic for the onset of diestrus. The day of estimated luteinizing hormone (LH) peak, considered as day 0, was calculated as 9 days before the onset of cytological determined diestrus [15, 16].

Forty-five days after the first clinical examination, the bitch showed serosanguinous vulvar discharge (considered as the first day of proestrus, day -8 prior to the expected LH peak) and cytological examination of a vaginal smear confirmed proestrus. Vaginal smears were repeated on days -7 and 0 and daily from day 2 to day 9. On day 4, cytological examination was characterized by a predominance of cornified epithelial cells. The bitch was then brought to a male of proven fertility and was mated once daily on day 4 and day 6. On day 18, ultrasound examination of the uterus showed three amniotic vesicles. One of them was resorbed on day 20 and the remaining two died on day 23.

Hematological and hematobiochemical parameters were within physiological ranges. For progesterone analyses, blood was collected in lithium heparin tubes and immediately centrifuged. Plasma samples were divided in two aliquots: one was used to determine progesterone levels with an in-house rapid semi-quantitative ELISA (BVT Ovulation^®^ test, Virbac Italia S.r.l., Milano, Italy) with four value ranges: 0–1, 1–2.5, 2.5–8 and >8 ng/mL. Using this kit, progesterone levels were >8 ng/mL on day 12, and 1–2.5 ng/mL on days 20, 23 (when embryo resorption occurred) and on day 26. The second aliquot was stored at −80 °C for later radioimmunoassay (RIA) analysis but the analysis failed due to technical problems.

The negative serological tests for infectious agents, the physiological hematological and biochemical parameters and the verification of pregnancy excluded infertility due to maternal illness or genital abnormalities and primary hypoluteoidism was therefore suspected based on extremely low plasma progesterone concentrations during early pregnancy.

In the next estrus after an interestrus interval of 5 months, the bitch was mated in the post-ovulatory period (day 3 to day 6 after the estimated LH peak). Plasma progesterone concentrations were >8 ng/mL until day 17 and 1–2.5 ng/mL on day 19. On the same day, transabdominal ultrasonography showed three apparently normal amniotic vesicles. However, further ultrasound examinations on days 20 and 22 indicated resorption of two out of three vesicles. Hematological and biochemical analyses revealed values within the physiological range. As hypoluteoidism was again suspected, the bitch was treated with 1.65 mg/kg progesterone in oil intramuscularly (PROGEST-E^®^, Fort Dodge Animal Health S.p.a., Bologna, Italy) daily from day 19 to day 22, and every 48 h from day 23 to day 58. Plasma levels of progesterone during this treatment were not measured. However, the efficacy of the treatment was evaluated through the assessment of fetal development monitored weekly from day 25 by ultrasonography. Normal fetal development was observed. This finding supported the suspicion of primary hypoluteoidism as cause of previous embryonic loss.

As signs of parturition were still absent on day 62, a Caesarean section was recommended, but the owner did not give the consent for surgery. On day 65, the bitch was brought to the VTH with signs of parturition. Dystocia due to fetal macrosomia was diagnosed and although a Caesarean section was immediately performed, the puppy died soon after birth.

**Fig. 1:**
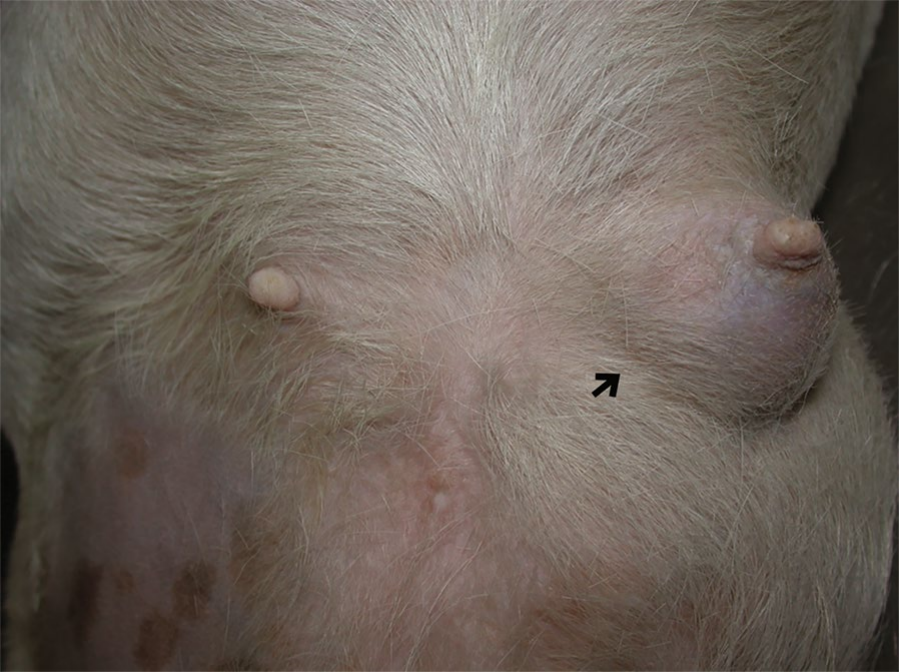
Gross appearance of the fibroadenoma in the 2nd left thoracic mammary gland (*black arrow*) at day 50 of the second pregnancy

**Fig. 2:**
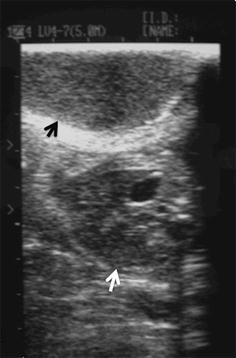
Ultrasound image of the mammary fibroadenoma and pregnant uterus (second pregnancy). A solid hypoechoic and heterogeneous area (*black arrow*) and parts of a fetus (*white arrow*) are seen

**Fig. 3:**
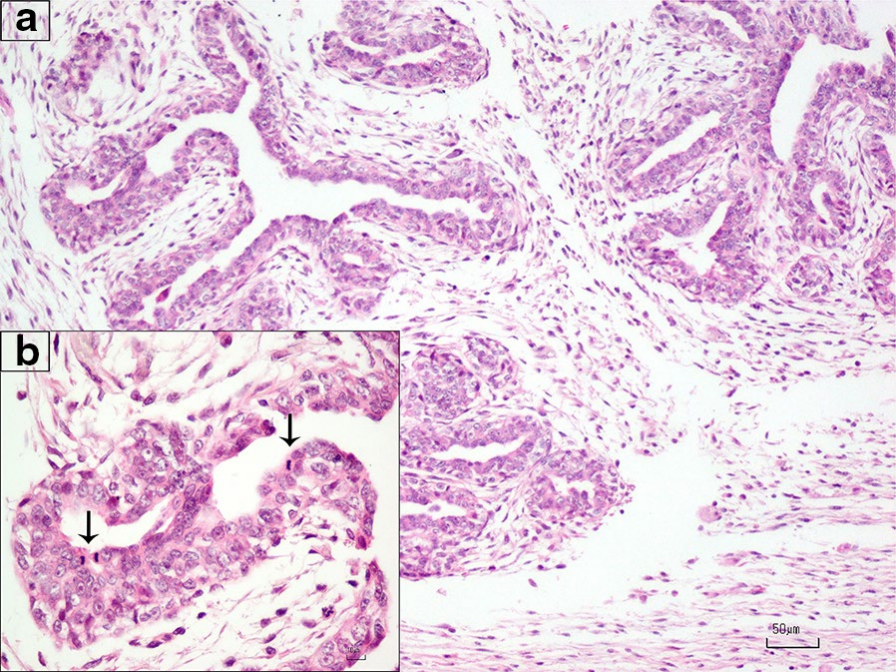
**a** Photomicrograph of the fibroadenoma. The neoplasm is characterized by epithelial cells arranged in ductal structures and surrounded by an edematous connective tissue. H&E. *Bar* = 50 µm. **b** Mitotic figures can be seen in the neoplasm (*arrows*). H&E. *Bar* = 10 µm

During the pregnancy, from day 32 to day 50, the 2nd left thoracic mammary gland (T2-L) increased in size up to a diameter of 6 cm (Fig. 1) ultrasonography revealed a hypoechoic and heterogeneous area (Fig. 2). On day 46, following local sub-cutaneous analgesia with lidocaine 2% (Ecuphar S.r.l., Milan, MI-Italy), a mammary biopsy was obtained by a semiautomatic 18G guillotine needle (Tsunami Medical, San Possidonio, MO-Italy), fixed in 10% neutral buffered formalin, processed, paraffin-embedded, sectioned at 3 µm and stained with hematoxylin and eosin (H&E) for histology. Microscopy revealed an expansive and moderately cellular neoplasm characterized by branching ductular structures lined by two to multiple layers of neoplastic cuboidal to columnar epithelial cells surrounded by a loose and edematous connective tissue (Fig. 3a). Epithelial cells of 15 µm in diameter with distinct cell borders had a moderate amount of homogeneous and eosinophilic cytoplasm and a central ovoid nucleus with a single eosinophilic nucleolus. Anisokaryosis and anisocytosis were moderate and mitotic figures ranged from 0 to 2 per high power field (Objective 40×; Fig. 3b). According to Misdrop et al. [17], these findings were consistent with those of a low-cellularity fibroadenoma. Colostrum was present in all mammary glands including the T2-L. Two months after Caesarean section, the mammary mass underwent spontaneous regression.

During the following estrus, after an interestrus interval of 6 months, breeding management and monitoring of proestrus–estrus were done as previously. On day-2, the bitch was in proestrus and was mated from day 4 to day 6 after the estimated LH-peak. Plasma progesterone concentrations were >8 ng/mL up to day 16 and 2.5–8 ng/mL on day 17. On days 18, 19, 40 and 51, the concentrations ranged from 1 to 2.5 ng/mL.

Since early primary hypoluteoidism was suspected in the past two pregnancies, the bitch received a peroral (PO) treatment with 0.075 mg/kg altrenogest (Regumate^®^, Intervet Italia S.r.l., Peschiera Borromeo, MI, Italy) on day 8 and then every 24 h until day 52 of pregnancy. The dose was reduced to 0.058 mg/kg PO from day 53 to day 57. Pregnancy was assessed by ultrasonography on days 18, 20 and 22, and five amniotic vesicles were detected at all times. Afterwards, weekly sonographic follow-up examination revealed normal embryonic and fetal development and viability. Recurrence of abnormal swelling of the T2-L occurred, reaching maximum size between days 43 and 50. The bitch whelped five live mature pups (two males and three females) on day 60. Colostrum was present in all glands including T2-L. Milk production was adequate and the pups were weaned at 2 months of age. The swelling of T2-L completely regressed within 3 months postpartum.

Three years later, the owner reported that the bitch was mated regularly during estrus, but she never showed signs of pregnancy nor treated with progestins again. The three daughters of the bitch never showed signs of estrus over the past 3 years. Furthermore, a short vagina that ended blindly just cranial to the urethral meatus and a clitoral hypertrophy were diagnosed in one of them.

## Conclusions

In clinical practice, it is difficult to assess whether abortion, fetal death or premature parturition may be the direct consequence of primary/true hypoluteoidism or occurring secondarily to infectious [5] or noninfectious [18, 19] causes affecting corpus luteum (CL) function (secondary hypoluteoidism). Hypoluteoidism is a poorly described condition in dogs [6, 7, 18] and there is limited evidence of abortion in bitches due to CL failure [1, 5, 8].

In the present case, hypoluteoidism was suspected on the basis of: (1) history of infertility; (2) clinical and gynecological findings; (3) plasma progesterone levels that dropped in three consecutive pregnancies at around day 20 after the LH peak; (4) pregnancy maintenance after progestogen supplementation.

The ELISA kit used for progesterone monitoring has excellent capacity in predicting the day of whelping in the bitch (91 versus 95% of RIA method) [20] and in the present case it corroborated the diagnosis of hypoluteoidism. However, we cannot totally exclude a deficiency of luteotropic pituitary support [21] as a cause of the hypoluteoidism, since levels of LH and prolactin were not assessed.

In the first progestogen unsupplemented pregnancy, plasma progesterone levels abruptly dropped causing embryonic death on days 2 to 5 after implantation. In the second and third monitored pregnancies, supplementation with either progesterone in oil or altrenogest resulted in a full term pregnancy. The time of last injection before delivery, the dosage and the duration of the treatment differed from protocols used by others. Purswell [6] administered progesterone (2 mg/kg) on days 50 and 53 after last breeding and the bitch whelped 4 days after the last administration. Hayer et al. [22] supplemented progesterone every 48 h from day 25 to 53 after ovulation and a Caesarean section had to be performed on day 64. In the present case, the bitch was treated for a longer period (i.e., from day 19 to day 58) with a lower dosage (1.65 mg/kg) and showed signs of delivery on day 65 after the LH peak, which correspond to 7 days after the last administration. It has previously been reported that following administration of exogenous progesterone, plasma progesterone concentrations return to basal levels within 72 h [6, 11, 23]. In this case, although signs of delivery were observed within the physiological time range, there was a delay of around 2 days on the expected date from the last progesterone administration, suggesting accumulation of exogenous hormone. Differences in delivery dates after the last administration of progestins may depend on the presence of undetected active corpora lutea. However, in the present case this can be ruled out considering the premature and abrupt drop in endogenous progesterone observed at around day 20 in all the investigated pregnancies.

It can be hypothesized that the delay in parturition may be responsible for the observed fetal macrosomia. This condition cannot be attributable to the singleton puppy syndrome, commonly characterized by delayed prepartum luteolysis due to insufficient fetal cortisol signaling [24]. In the reported case, active corpora lutea were absent and lack of luteolytic response to fetal signals could be excluded.

In the third pregnancy, treatment with altrenogest was performed and the bitch whelped spontaneously 3 days after the last administration. Other authors reported treatments with altrenogest for only 4 days at similar doses, with whelping 2–4 days after the last administration [7, 18].

Besides progesterone in oil [10, 11, 23] and altrenogest [7, 9, 18], medroxyprogesterone acetate (MPA; 0.1 mg/kg, PO) has been suggested for supplementary treatment of luteal deficiency [1, 8]. Medroxyprogesterone acetate administered to maintain pregnancy may cause masculinization of female fetuses and reduce milk production in the first 3 days after parturition [1]. However, after oral MPA application starting later than day 30 post-ovulation or when organogenesis is completed, no congenital abnormalities have been reported [8]. In the present case, the owner reported that the daughters of the bitch treated with altrenogest have never exhibited signs of estrus, suggesting abnormalities of the ovaries, and that in one of them, an enlargement of the clitoris and a short vagina were present. Such abnormalities could be related to progestin administration during the embryonic stage, interfering with normal development of the genital tract. If progestins are used as supplemental treatment in hypoluteoidism, the administration should not start before days 30–35 in order to avoid genital abnormalities [8, 25]. Since hypoluteoidism is an ovarian dysfunction and its treatment with progestins might lead to severe unwarranted effects, it is preferable to exclude the affected bitches from breeding.

In contrast with previous findings, the administration of altrenogest did not affect lactogenesis and did not reduce milk production [1, 7]. However, following treatment with progesterone in oil or altrenogest, the bitch developed a mammary low-cellularity fibroadenoma showing similar morphologic structures as feline fibroadenomas [17]. Based on the similarities of the present case and previously reported feline cases i.e., correlation of progestin stimulus with gland hyperplasia and spontaneous regression after the end of the hormonal treatment [13, 14, 17], this lesion should be classified as a fibroadenomatous change according to the current histological classification of canine mammary tumors [26]. This lesion is a progesterone-dependent mammary gland hyperplasia found in young pregnant or pseudopregnant cats in one or multiple glands and in female or male cats treated with progestins [13, 14, 17, 27]. Spontaneous regression is usually observed after luteolysis, ovariectomy [27] or anti-gestagenic treatment [28]. Although the lesion is assumed to occur only in cats, fibro-epithelial tumors with stromal proliferation and similar morphologic features have been reported in mammary glands of bitches exposed to aerosolized cyclosporine [29].

In summary, the semi-quantitative ELISA kit used for analyzing progesterone in plasma proved a reliable aid to detect early hypoluteoidism before the onset of embryonic death. The diagnosis of hypoluteoidism is strongly supported by the decrease in plasma progesterone concentration before embryonic loss and by the maintenance of subsequent pregnancies under administration of progesterone in oil or altrenogest. Altrenogest might be an alternative to progesterone for maintaining pregnancy to term. However, the use of altrenogest is inappropriate during early pregnancy due to the high risk of interfering with the embryonic development of the genital tract. Moreover, as described in the present case, progestogen treatments may lead to mammary gland neoplasia in predisposed individuals. Apart from these potential severe unwarranted effects, it must be considered that hypoluteoidism may have a genetic component. Since a fertility suppressing effect within a breed cannot be excluded, bitches having this condition should be excluded from breeding.
